# Quantitative Assessment of Hydrological Alteration Caused by Irrigation Projects in the Tarim River basin, China

**DOI:** 10.1038/s41598-017-04583-y

**Published:** 2017-06-27

**Authors:** Lianqing Xue, Hui Zhang, Changbing Yang, Luochen Zhang, Chao Sun

**Affiliations:** 1 Hydrology and Water Resources College, Hohai University, Nanjing, 210098 P. R. China; 2 College of Water & Architectural Engineering, Shihezi University, Shihezi, 832003 P. R. China; 3 Bureau of Economic Geology, Jackson School of Geosciences, University of Texas at Austin, Austin, 78712 USA; 4 Tarim River Basin Administration, Korla, 841000 P. R. China

## Abstract

The Tarim River is the longest inland river at an arid area in China. Deterioration in its ecohydrological system has received much attention world widely. This study presents quantitative assessment of hydrological alterations in the hydrological regime of the Tarim River caused by reservoir irrigation and channel irrigation over a period of over a half century. The improved indicators of hydrologic alteration and range of variability approach were applied to the daily flow rates at the two representative hydrological stations. Our study shows that the annual extreme water conditions (1-, 3-, 7-day annual minimum and extreme low timing) have been altered, compared with the pre-impact period. The average flow rate in July, the 30-day annual maximum flow rates, the date for the maximum rate, the rise rate, and the fall rate show a significant decreasing trend. The improved overall degree of hydrological alteration for the two stations are approximately 68.7% and 61.8%, suggesting a high degree of alteration. This study greatly improved our understanding of impacts of irrigations on the ecohydrological characteristics in the Tarim River.

## Introduction

Shortage in freshwater resources has become one of the prominent issues in many regions of the world, especially in developing countries, such as China^1, 2^. With economic development, demand in freshwater resources is increasing continuously and posing tremendous pressure on water resources management. In the past, development of water resources projects at various scales has been considered a solution strategy for mitigating shortage in water supply, especially, in Northwestern China where water division projects have been built on most major rivers over the last half century^3, 4^. Because the water needs of humans and natural ecosystems are generally considered as competing with each other, the over usage of water by humans could lead to severe alterations to the ecohydrological regimes of a river system. Many examples of ecological consequences of altered natural flow regimes have been reported in literature^5–7^.

Many efforts have been devoted to quantitatively assess the anthropogenic impacts on water environments with more than 170 hydrologic metrics to describe different components of flow regime and characterize the ecologically relevant attributes^8–10^. In the last two decades, the most commonly used hydrologic indices for characterizing flow regimes are the Indicators of Hydrologic Alteration (IHA)^11–13^. The IHA includes 32 parameters which can be categorized into five groups of hydrologic features, covering a full range of natural flow variability, from magnitude, frequency, timing, duration to rate of change^14, 15^. A comprehensive review conducted by Olden and Poff^9^ concluded that the IHA can adequately characterize flow regimes with ecological knowledge and greatly improve our understanding of the interactions between flow regimes and riverine ecosystems. The Range of Variability Approach (RVA), proposed by Richter *et al*.^16^, is a widely used approach for quantitatively assessing the alteration of flow regime by comparing frequency distributions of IHA parameters during the pre-impact and post-impact^17–20^. The river managers try to evaluate potential hydrologic alterations within a targeted range which is related to the natural range of variability in parameters. Each IHA has the range of variability which can be determined based on the selected percentile thresholds or a simple standard derivations by the 25^th^ and 75^th^ percentiles of the pre-impact IHA values.

The primary objective of this study is to assess hydrological alterations of flow regimes in terms of 32 IHAs with the RVA due to water division projects for irrigation at the Tarim River Basin. The TRB is located in the northwest arid area of China, exhibiting a fragile ecological environment^21, 22^. The Tarim River is the longest inland river in China with a mainstream of 1321 km in length. Over the last half century, because of intensive human interventions, such as water exploitation and utilization with various water division projects, the natural ecological processes have experienced severe changes and the significant imbalance in the natural distribution of water resources has become one of great concerns by public^23^. Many studies about impacts of human activities and climate change on the ecological and water systems at the TRB were reported^24–40^. Chen *et al*.^41^ presented a study about desiccation of the Tarim River showing an increasing trend in length affected and time duration and concluded that water transfer to the lower reach will be severely impacted if the tendency is not constrained and the downstream “Green Corridor” protection will be severely impacted. Qi *et al*.^42^ reported that intensive anthropogenic disturbance has been one of the foremost factors leading to deterioration of water resources in the region. Tao *et al*.^43^ evaluated ecohydrological responses to water diversion in the lower reaches of the Tarim river using groundwater wells and vegetation as main indicators. However, up to date there are very few studies reported on quantitative assessment of hydrologic alterations of flow regimes in the Tarim River due to water diversion projects for irrigation at the basin. Sun *et al*.^44^ analyzed long streamflow series collected from 5 hydrological stations in the Tarim River in terms of the only one hydrological parameter, the 7-day low flow. Late, Wang *et al*.^22^ assessed streamflow changes in the Tarim River basin based on daily data collected at 7 hydrological stations, but focused on trends in hydrological extremes, including magnitude, duration and high flow frequency. In this study, the extended streamflow data during 1957 through 2014 were comprehensively assessed in terms of 32 IHAs to characterize hydrological alterations between pre-impact and post-impacts of water diversion projects. In addition, the post-impacts were further divided into two phases: the reservoir irrigation and channel irrigation. To the best of our knowledge, the study presented here is the first kind of comprehensive assessment on hydrological alterations of flow regimes in the Tarim River.

## Results

### Impacts on the magnitude of monthly streamflow

**Figure 1:**
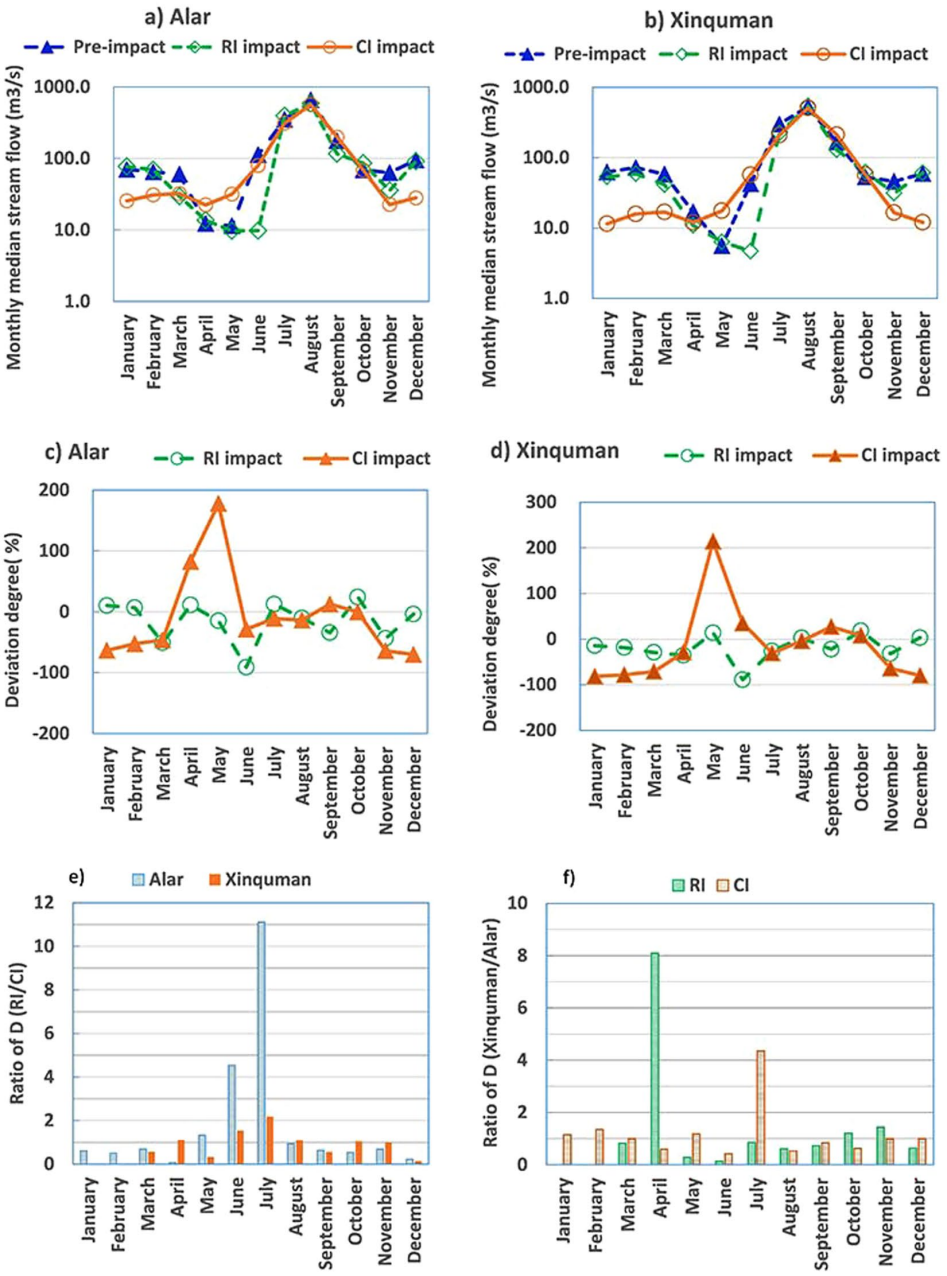
Plots of median monthly stream flow at (**a**) the Alar station and (**b**) at the Xinquman station, deviation degree of median monthly stream flow (**c**) at the Alar station and (**d**) at the Xinquman station, ratio of degree of hydraulic alteration (**e**) for the RI impact to the CI impact, and (**f**) between the two stations (note: RI for reservoir irrigation from 1973 to 1989 and CI for channel irrigation from 1995 to 2014).

The median value, deviation degree and degree of alteration for the monthly streamflow of the Group 1 IHAs at the Alar station and the Xinquman station are listed in Table 1. Deviation degree and degree of alteration for each IHA were calculated with Equations (1) and (2). At the Alar station, except in January, February, April, and July, the stream flow in the other months decreased from the pre-impacted period to the RI impacted period (Table 1). The most significant decrease in the stream flow occurred in June, from ~113 m^3^/s during the pre-impacted period to ~10 m^3^/s during the RI impacted period (Fig. 1a). At the Xinquman station, the RI and CI impacts on median value of monthly stream flow show a similar pattern at the Alar station (Fig. 1b). At the Alar station, during the RI impact period the deviation degree (*P*) for 7 out of 12 months is less than 0 while during the CI impacted period deviation degree for 9 out of 12 months is a negative value, suggesting the that both reservoir irrigation and channel irrigation have decreased the stream flow rate in the Tarim River, compared to the natural condition during the pre-impacted period. At the Xinquman station, deviation degree is less than 0 for 8 out 12 months during both the RI and CI impacted periods (Fig. 1d).

**Table 1:** Comparison of hydraulic alterations in Group 1 IHAs (monthly streamflow) at the two stations for the pre-impact, RI impact and CI impact.

Indicators	The Alar station							The Xinquman station						
	Pre-impact	RI impact			CI impact			Pre-impact	RI impact			CI impact		
	M	M	P (%)	D (%)	M	P (%)	D (%)	M	M	P (%)	D (%)	M	P (%)	D (%)
January	70.1	77.2	10	54	25.5	−64	87	62.3	53.4	−14	0	11.5	−82	100
February	65.0	69.3	7	38	30.8	−53	74	72.3	58.9	−18	0	15.9	−78	100
March	60.2	29.6	−51	69	32.2	−47	100	58.1	41.3	−29	57	16.9	−71	100
April	12.3	13.7	11	7	22.4	82	87	16.9	10.9	−36	57	12.0	−29	51
May	11.4	9.7	−15	99	31.7	178	74	5.6	6.4	14	29	17.6	214	88
June	112.5	9.8	−91	100	80.1	−29	22	42.4	4.7	−89	14	57.1	35	9
July	350.0	394.0	13	100	311.5	−11	9	300.0	223.0	−26	86	208.4	−31	39
August	657.0	589.0	−10	69	565.5	−14	74	530.0	544.0	3	43	509.0	−4	39
September	176.9	116.0	−34	39	199.0	12	61	166.5	129.5	−22	29	211.5	27	51
October	68.9	85.6	24	24	68.6	0	43	53.0	62.6	18	29	57.3	8	27
November	63.2	35.6	−44	69	22.7	−64	100	45.9	31.4	−32	100	16.6	−64	100
December	94.6	91.0	−4	22	28.1	−70	100	59.3	61.2	3	14	12.1	−80	100

Note: M for median value, P for deviation degree estimated using Equation (1), D for degree of hydrological alteration estimated with Equation (2), RI for Reservoir irrigation, and CI for Channel irrigation.

At the Alar station, calculation of degree of hydrological alteration (D) for monthly stream flow shows that 3 monthly stream flows fall within the category of low alteration (D < 33%), 3 within the category of moderate alteration (33% < D < 67%) and 6 within the category of highly alteration (D > 67%) during the RI impacted period while 2 within the category of low alteration, 2 within the category of moderate alteration and 8 within the category of highly alteration during the CI impacted period (Table 1). At the Xinquman station, 2 monthly stream flows (July and November) are highly altered with D > 67% during the RI impacted period while 6 monthly stream flows are highly altered during the CI impacted period. Figure 1e shows comparison between the impacts of reservoir irrigation and channel irrigation on the monthly stream flow in the Tarim River in terms of D ratio (=D during the RI impacted period/D during the CI impacted period). If D ratio is greater than 1, this suggests that RI has more significant impacts on monthly stream flow than CI. Obviously the D ratios in June and July are greater than 1 at both stations (Fig. 1e), suggesting that RI has stronger impacts on the monthly stream flows than CI does. On the other hand, D ratios in December at both stations are close to 0.2, indicating that CI has more significant impacts on the monthly stream flow than RI does.

Degree of hydrological alteration of the monthly stream flow at the Alar station is also compared to that at the Xinquman station in terms of their ratio, D during the RI (or CI) impacted period at the Xinquman station divided by D during the RI (or CI) impacted period at the Alar station. If the ratio > 1, this indicates that the post impact (either RI or CI) on the monthly stream flow is more significant at the Xinquman station than at the Alar station. Apparently, reservoir irrigation has more significant impact on the monthly stream flow in April with a ratio up to 8 and channel irrigation has more significant impact on the monthly stream flow in July with a ratio up to 4 at the Xinquman station than at the Alar station. In June, both RI and CI have stronger impact on the monthly stream flow at the Alar station than at the Xinquman station (Fig. 1f). The results show clearly that the impacts of RI and CI on monthly runoff are significant, and the operation of reservoirs could decrease the magnitude of runoff.

### Hydrologic alteration for the annual extreme flow conditions

Table 2 lists median value, deviation degree and degree of hydraulic alteration for the annual extreme flow conditions including 11 IHAs in the Group 2 and 2 IHAs in the Group 3 at the Alar station and the Xinquman station.

**Table 2:** Comparison of hydraulic alterations in extreme flow conditions (Groups 2 and 3) at the two stations for the pre-impact, RI impact and CI impact.

Indicators	The Alar station							The Xinquman station						
	Pre-impact	RI impact			CI impact			Pre-impact	RI impact			CI impact		
	M	M	P (%)	D (%)	M	P (%)	D (%)	M	M	P (%)	D (%)	M	P (%)	D (%)
1-day minimum	5.3	6.0	13	49	11.7	120	100	2.0	2.6	33	43	2.4	22	39
3-day minimum	5.4	6.0	10	39	12.0	120	74	2.0	2.7	36	29	2.5	25	27
7-day minimum	5.5	6.2	13	24	12.2	124	87	2.1	2.8	34	29	2.6	22	15
30-day minimum	9.3	7.6	−18	39	16.6	79	100	4.5	5.1	13	71	5.1	13	34
90-day minimum	30.5	13.9	−55	7	23.5	−23	69	18.1	10.2	−44	14	8.7	−52	3
1-day maximum	1090	1330	22	24	1170	7	17	1020	863.0	−15	14	969.5	−5	64
3-day maximum	1013	1267	25	24	1047	3	4	1008	821.3	−19	0	897.4	−11	27
7-day maximum	944.1	1123	19	8	962	2	9	832.1	765.7	−8	0	841.1	1	3
30-day maximum	726.0	773	6	85	669	−8	61	603.2	566.2	−6	29	592.9	−2	3
90-day maximum	446.8	430.4	−4	69	425.3	−5	87	400.3	342.1	−15	43	357.4	−11	64
Base flow index	0.032	0.051	60	7	0.102	218	100	0.016	0.024	55	43	0.02	57	3
Julian date of minimum	143	163	14	24	184	29	48	159	176	11	71	148	−7	88
Julian date of maximum	226	220	−3	39	217	−4	9	226	222	−2	14	218	−4	27

Note: M for median value, P for deviation degree estimated using Equation (1), D for degree of hydrological alteration estimated with Equation (2), RI for Reservoir irrigation, and CI for Channel irrigation.

**Figure 2:**
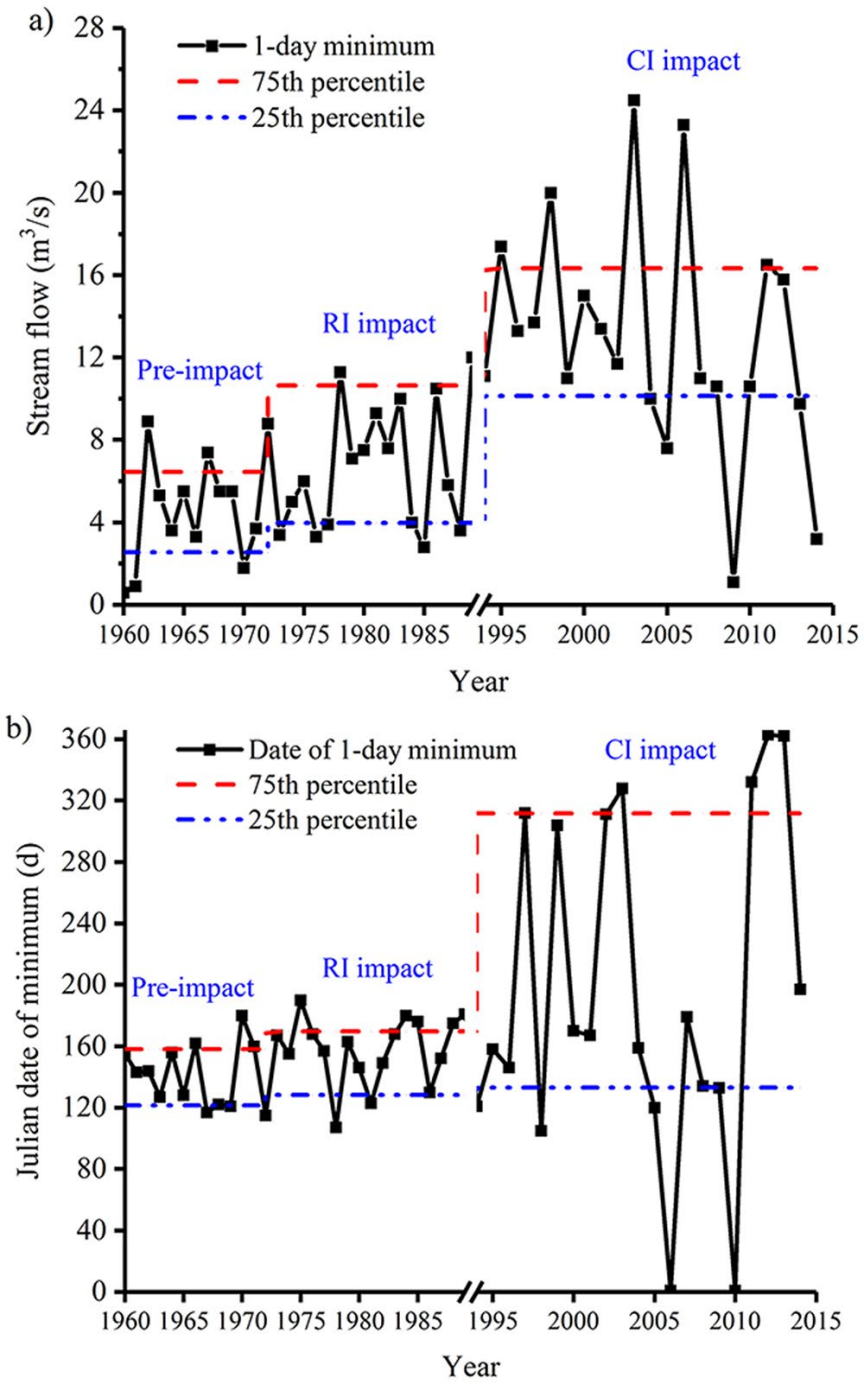
Plots of (**a**) 1-day minimum stream flow and (**b**) Julian date of minimum stream flow occurrence at the Alar station.

**Figure 3:**
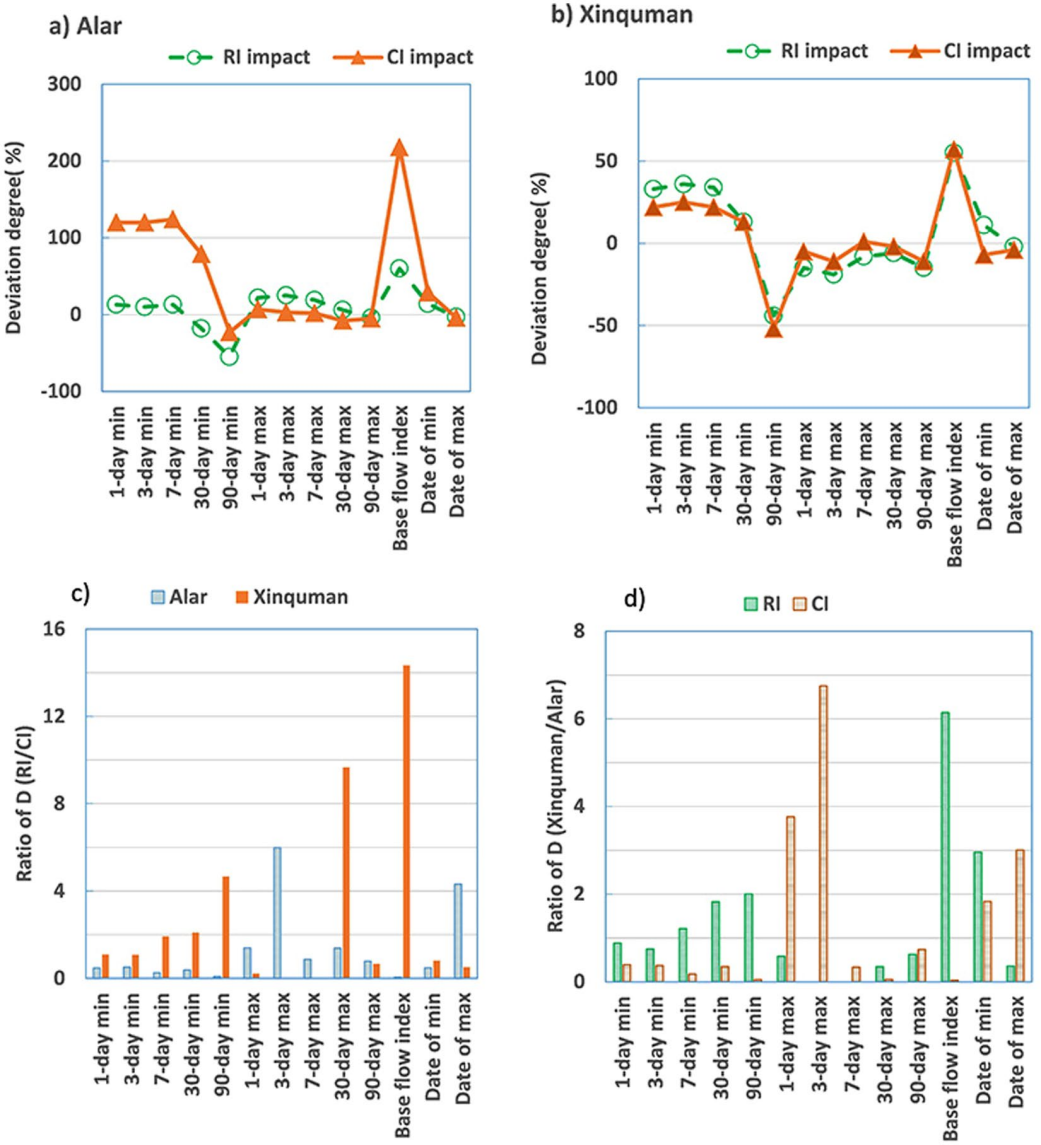
Plots of deviation degree of IHAs in Groups 2 and 3 at (**a**) the Alar station and (**b**) the Xinquman station, and ratio of degree of hydraulic alteration for (**c**) the RI impact to the CI impact and (**d**) the Alar station to the Xinquman station (note: RI for reservoir irrigation from 1973 to 1989 and CI for channel irrigation from 1995 to 2014).

Figure 2 illustrates impacts of RI and CI on the annual extreme flow conditions in the Tarim River at the Alar station. It seems that the 1-day minimum was elevated by 13% during the RI impacted period and 120% during the CI impacted period at the Alar station (Fig. 2a). However, the date with 1-day minimum stream flow was slightly delayed (~20 days) during the RI impacted period and significantly delayed (~40 days) during the CI impacted period at the Alar station (Fig. 2b). At the Alar station, 9 out of 13 IHAs have a positive deviation degree during both the RI and CI impacted periods. The base flow index has a highest deviation degree, up to ~220% during the CI impacted period (Fig. 3a). At the Xinquman station, 6 out of 13 IHAs have a positive deviation degree during both the RI and CI impacted periods. Similar to that at the Alar station, the base flow index has the maximum deviation, up to ~57% for both the RI and CI impacted periods (Fig. 3b), suggesting that the date with minimum stream flow was likely postponed from the late of April to early June.

At the Alar station, 7 IHAs (7-, 90-day minimum, 1-, 3-, 7-day maximum, base flow index, Julian date of minimum) show low alterations with D < 33% and 2 IHAs (the 30-, and 90-day maximum) show highly alterations with D > 67% during the RI impacted period. However, during the CI impacted period, 7 IHAs show highly alterations with D > 67% (Table 2). At the Xinquman station, during the RI impacted period 2 IHAs (Julian date of minimum and 30-day minimum) show highly alterations with D > 67% and during the CI impacted period only one IHA (Julian date of minimum) shows highly alteration. Figure 3c shows the D ratio of each IHA among the Groups 2 and 3 between the RI impacted and the CI impacted periods at the two stations. At the alar station, 4 IHAs have a D ratio greater than 1 while at the Xinquman station, 7 IHAs have a D ratio greater than 1, suggesting that compared to CI, RI has stronger impacts on the extreme flow conditions at the Xinquman station than at the Alar station (Fig. 3c). Figure 3d shows comparison between the two stations in terms of degree of hydraulic alteration. Obviously, RI has more significant impacts on the 7-, 30-, 90-day minimum, base of index and date of minimum at the Xinquman station than at the Alar station. CI altered 1-, 3-day maximum, date of minimum, and date of maximum more significantly at the Xinquman station than at the Alar station.

### Hydrologic alteration of frequency and duration of high and low pulses

**Figure 4:**
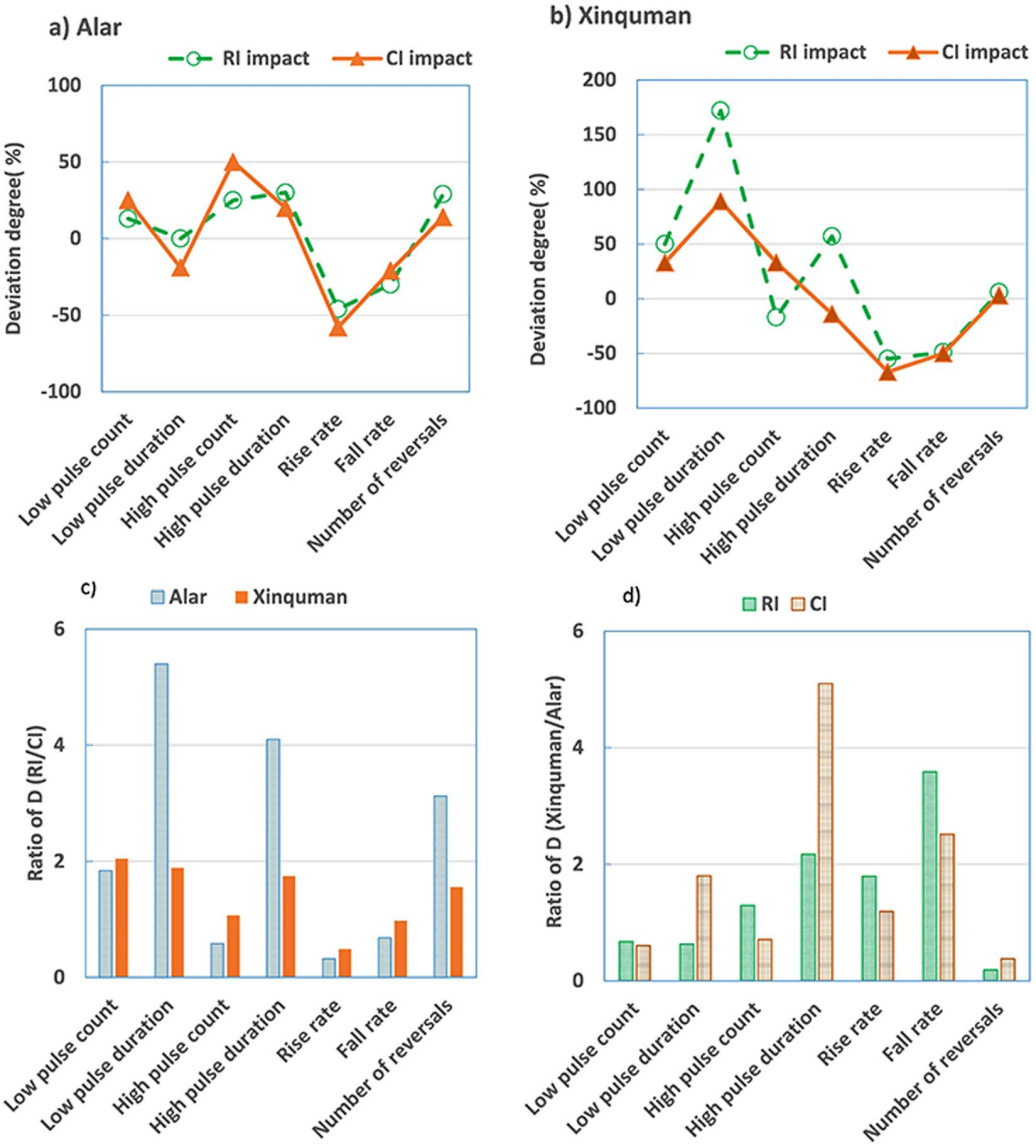
Plots of deviation degree of IHAs in Groups 4 and 5 at (**a**) the Alar station and (**b**) the Xinquman station, and ratio of degree of hydraulic alteration for (**c**) the RI impact to the CI impact and (**d**) the Alar station to the Xinquman station (note: RI for reservoir irrigation from 1973 to 1989 and CI for channel irrigation from 1995 to 2014).

Assessment of impacts of RI and CI on the indicators in Group 4 in terms of deviation degree and degree of hydraulic alteration is listed in Table 3. At the Alar station, both RI and CI show similar impacts on the frequency and duration of high and low pulses in terms of their deviation degree (Fig. 4a). However, at the Xinquman station, the low pulse duration has a much higher deviation (P = ~170%) during the RI impacted period than P (~90%) during the CI impacted period. (Fig. 4b). During the period of RI, the Alar station experienced a slightly increase in the low pulse counts, with the duration of low pulses remains unchanged (Table 3). The high pulses exhibited a positive increase in the counts and duration. During the CI impacted period, both the hydrologic stations show an increase in the number of low pulse counts and high pulse counts (Table 3). As the number of low pulse counts increasing, a dry and wet cycle will worsen the ecological natural development at the Tarim River floodplain. The moderate increase in the duration of high pulses may favor the riverine ecosystem. Because the increase of high pulse duration brings enough nutrients to the plants (especially the poplar) and animals along the river bank, and it may play a positive role in promoting the development of river biodiversity.

**Table 3:** Comparison of hydraulic alterations for indicators in Groups 4 and 5 at the two stations for the pre-impact, RI impact and CI impact.

Indicators	The Alar station							The Xinquman station						
	Pre-impact	RI impact			CI impact			Pre-impact	RI impact			CI impact		
	M	M	P (%)	D (%)	M	P (%)	D (%)	M	M	P (%)	D (%)	M	P (%)	D (%)
Low pulse count	4.0	4.5	13	70	5.0	25	38	3.0	4.5	50	47	4.0	33	23
Low pulse duration	13.0	13.0	0	54	10.5	−19	10	9.0	24.5	172	34	17.0	89	18
High pulse count	4.0	5.0	25	24	6.0	50	41	3.0	2.5	−17	31	4.0	33	29
High pulse duration	5.0	6.5	30	41	6.0	20	10	11.0	17.3	57	89	9.5	−14	51
Rise rate	5.0	2.7	−46	24	2.1	−58	74	3.4	1.5	−55	43	1.1	−67	88
Fall rate	−3.0	−2.1	−30	24	−2.4	−21	35	−2.1	−1.1	−49	86	−1.1	−50	88
Number of reversals	69.0	89.0	29	75	78.5	14	24	64.0	68.0	6	14	66	3	9

It seems that RI has more significant impact on the 4 indicators of Group 4 than CI does at both the Alar station and Xinquman station (Fig. 4c). CI and RI have much more significant impacts on the high pulse duration at the Xinquman station than at the Alar station (Fig. 4d).

### Hydrologic alteration of rate and frequency of flow conditions change

Assessment of the Group 5 IHAs is listed in Table 3. Deviation degrees of rise rate and fall rate are negative at the two stations for the period of both RI and CI impacted periods, suggesting that both indicators were statistically reduced. On the other hand, deviation degrees of number of reversals are positive at the two stations for the period of both RI and CI impacted periods (Table 3). Note that deviation degrees of the Group 5 IHAs are very similar during the RI and CI impacted periods (Fig. 4a,b). At the Alar station, the number of reversals shows highly alteration (D = 75%) during the RI impacted period and low alteration (D = 24%) during the CI impacted period (Table 3). At the Xinquman station, both rise rate and fall rate show highly alteration during the CI impacted period. It seems that RI has more significant impacts on number of reversals at both stations than CI does (Fig. 4c). Both RI and CI show stronger alterations on the fall rate at the Xinquman station than at the Alar station.

**Figure 5:**
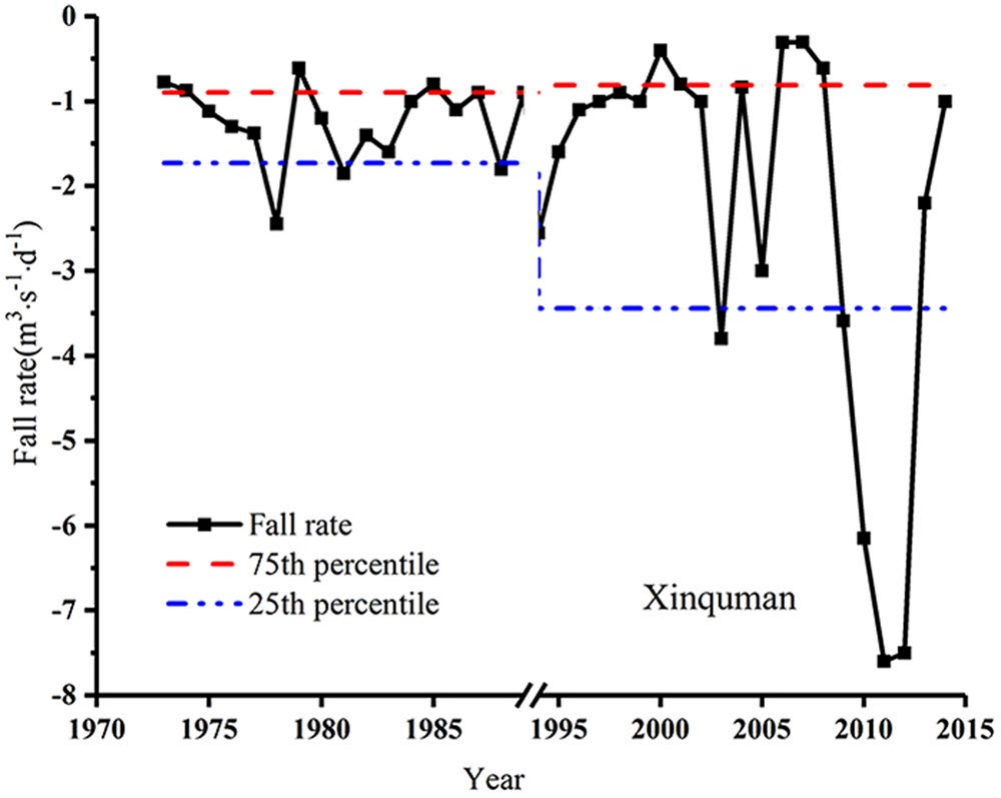
The average flow reduction rate during 1973 to 2014 at Xinquman station.

Because of channel irrigation, fall rate of flow rate at the Xinquman station showed a significant increase trend from 2009 through 2012, even beyond the 25^th^ percentile (Fig. 5), suggesting that more actions should be taken to prevent deterioration in the ecohydrological system of the Tarim river because frequent fluctuation in the streamflow may destroy the stability of animal and plant habitats.

### Overall degree of hydrologic alteration

**Figure 6:**
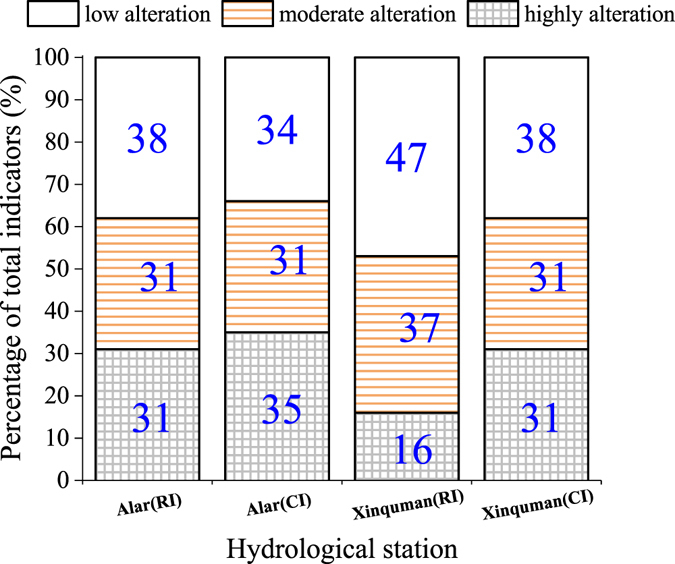
Bar plot of percentage of IHAs that are categorized into three ranks during the RI impacted period and the CI impacted period at the Alar and Xinquman stations.

Overall degree of hydrological alteration is shown in Fig. 6 with a proportion chart of IHAs at two hydrological stations at the two impacted periods: RI and CI. It can be seen that 34% to 48% of 32 indicators show low alteration, 31% to 37% with moderate alteration and 16% to 35% with highly alteration. This finding suggests the spatial-temporal hydrologic alterations in the Tarim River: 1) the Xinquman is further to the irrigation projects and has a smaller percentage of hydrological indicators with highly alteration and 2) CI has more impacts than RI, especially at the Xinquman station (Fig. 6).

Table 4 summarizes overall degree of hydrological alteration estimated for each group. The DHA of Group 1 indicators (the monthly flow rate) shows highly alteration with D > 65% during the RI impacted period. The DHA of the Group 2 varies from 35% to 41.8% at the Alar and Xinquman stations. During the RI impacted period (1973–1989), the ODHA reached 52.8% and 46.4% at the Alar and Xinquman stations, both showing moderate alterations in the ecohydrologic conditions. The improved ODHA (IODHA) are 60.3% at the Alar station and 55.4% at the Xinquman station, higher alterations calculated with Equation (4), suggesting that ODHA may underestimate the degree of hydrologic alteration. During the CI impacted period (1995–2014), the IODHA are 68.7% at the Alar and 61.8% station at the Xinquman station.

**Table 4:** Overall degree of hydrological alteration in the Tarim River for the RI impact and the CI impact.

	hydrologic station	Degree of Hydrologic Alteration (%)					ODHA	IODHA
		Group 1	Group 2	Group 3	Group 4	Group 5		
RI Impact	Alar	65.3	41.8	32.1	50.2	47.1	52.8(M)	60.3(H)
(1973–1989)	Xinquman	48.8	35.0	51.5	55.2	55.9	46.4(M)	55.4(M)
CI Impact	Alar	75.1	73.5	34.5	28.8	49.3	66.2(H)	68.7(H)
(1995–2014)	Xinquman	74.7	33.7	65.0	32.8	71.9	58.0(M)	61.8(H)

Note: ODHA represents the overall degree of hydrological alteration estimated with Equation (3) and IODHA is for the improved overall degree of hydrological alteration estimated with Equation (4). For each group, the degree of hydrologic alteration (DHA) was estimated using Equation (3).

### The impact of climate change on the flow regime of the Tarim River

**Figure 7:**
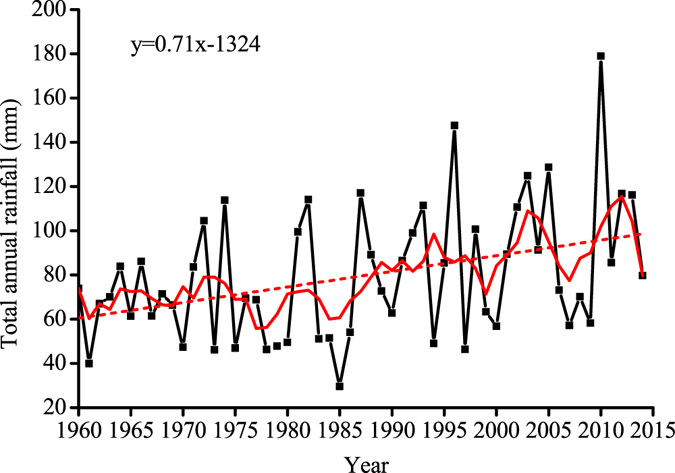
Temporal trend in annual precipitation at the upper reach of the Tarim River over 55 years (dash line is the linear fitting for the annual precipitation over 55 years with a slope of 0.71).

A hydrological flow regime may be altered by both human activities and climate change. In order to investigate impacts of climate change on the hydrological alterations, the precipitation data over the period of 1960 through 2014 from ten hydrological stations were analyzed. The annual precipitation at the upper reach of the Tarim River shows an overall increasing trend during the last 55 years, suggesting that climate change has a positive impact on the streamflow of the Tarim River (Fig. 7). Such an increase in the annual streamflow series at the up reach of the Tarim River is likely due to the fact that the increasing temperature at the study area which results in melting of more snow and ice at the mountainous area.

## Discussion

This study presents assessment of hydrological alterations in the flow regime of the Tarim River over the past five decades in terms of 32 IHAs which have been commonly used to characterize an ecohydrological system. Our study confirms that the average monthly flow in Tarim River was greatly altered by human activities because of reservoir irrigation and channel irrigation. The average monthly flow rate decreased in most months, especially in March, June and August. At both the Alar and Xinquman stations, there was little change in the maximum monthly flow rate and the obvious increase in the minimum runoff was caused by reservoir irrigation. The date with a minimum runoff was delayed from late April to early May at the Alar station. The number of low pulse counts were increased. The number of reversal times were increased at the Xinquman station.

The impact of the reservoirs on the flow regime is more significant at the Alar than at the Xinquman due to the different spatial distribution. The overall degree of hydrologic alteration at Alar and Xinquman are 60.3% and 55.4% respectively. During the CI impacted period from 1995 through 2014, the overall degree of hydrologic alteration at Alar and Xinquman are 68.7% and 61.8% respectively, which are both belong to highly alteration. Xinquman station, considering influence of interval water division irrigation projects, always exhibits a moderate alteration than the Alar station.

The relationship between hydrological processes and ecological system is tightly influenced by human-induced stresses such as reservoir construction, farmland irrigation, urban water supply and flood control. The well-known streamflow characteristics including hydrologic event magnitude, frequency, timing, duration and rates of change are often closely connected to river habitat variation. According to the response mechanism of ecological system reflected by the above mentioned characteristics, the effects of water conservancy projects on the ecological system in the TRB are mainly summarized as follows: (1) The reduction of streamflow in most months is likely contributing to the frequent change of drought and wet in different seasons. The decrease of summer flow in several days may leads to negative effects on the aquatic habitat, migratory and reproductive for fish species in downstream channel. (2) The reduction in 1-day maximum flow at the Xinquman station is likely restricting the nutrients transportation corridor between riverbed and floodplains. (3) Julian date of minimum streamflow lagged 41 days behind the nature state at the Alar station, which may threaten the riverine living environments, including the surrounding vegetation and aquatic organisms.

With the aim to storage water in flood season and reduce flood disaster reasonably, some typical reservoirs and lots of diversion plots are constructed and operated sequentially, which is a driving force of hydrologic regime in riverine ecosystems. Our study clearly shows that inappropriate management of water conservancy projects could dramatically alter the hydrological regime of the Tarim River. Our results presented in this study may provide a guidance in designing strategies to recover the ecohydrological system of the Tarim River, as well as those rivers at arid areas in the world.

## Materials and Methods

### Study area

**Figure 8:**
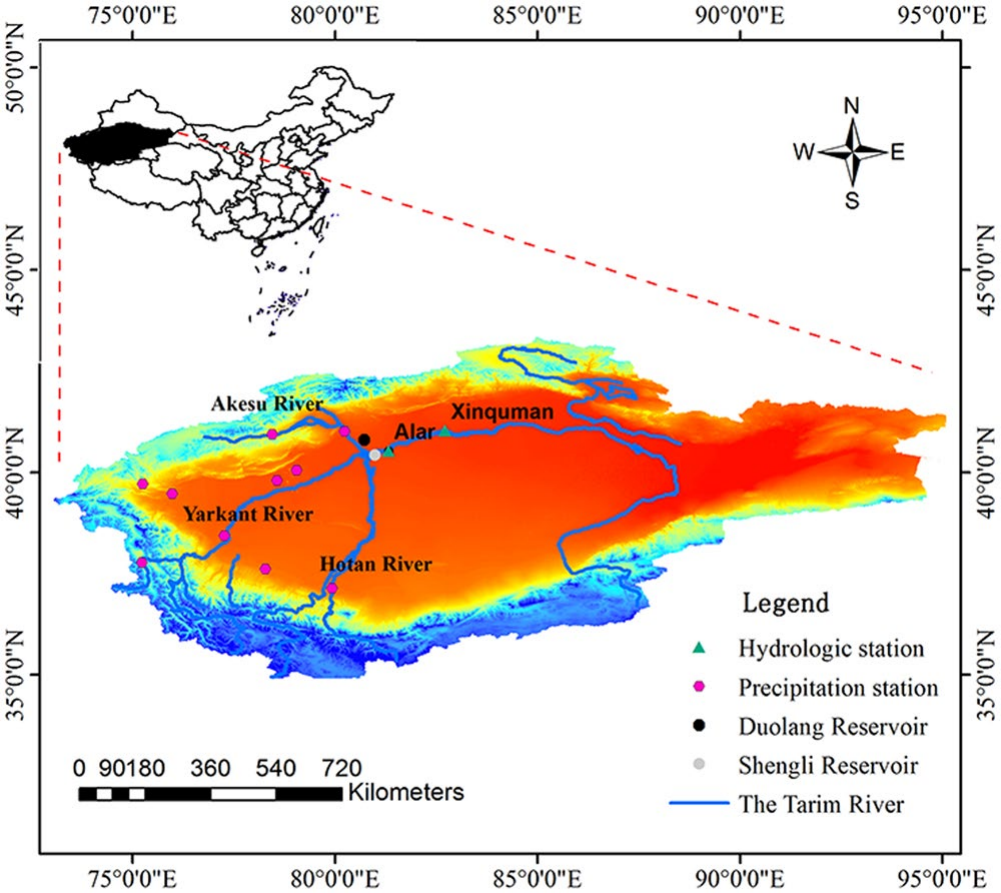
Sketch map of the Tarim River Basin and locations of hydrologic stations. (The map was generated with data available from the Chinese Geospatial Data Cloud using ESRI’s ArcGIS (version 10.1; http://www.gscloud.cn/)).

The Tarim River, located in the southern Xinjiang Province, is the longest inland river in China. Its mainstream begins from the west of the junction of Yarkant River, Hotan River and Akesu River and empties to the Taitema Lake^37^, with a total length of 1321 km, as shown in Fig. 8. The Tarim River is supplied by ice and snow melting and precipitation in the mountains with a drainage area of approximately 1.02 million km^2^. The Tarim Basin has an extreme drought desert climate. The average annual temperature is about 10.6 °C to 11.5 °C with monthly mean temperature ranging from 20 °C to 30 °C in July and −10 °C to −20 °C in January^26^. Compared to the average annual precipitation of 200–300 mm in the mountainous headwater regions^37^, the average annual precipitation at the mainstream area of the Tarim River is only about 50–80 mm. The annual potential evaporation ranges from 2000 mm to 2900 mm.

Agriculture is the main economic activity at the Tarim River Basin and heavily relies on various water diversion projects for irrigation in the Tarim River. The water diversion projects for irrigation include two main types: reservoir irrigation and channel irrigation. The reservoir irrigation is mainly to transport water through diversion canals and store in plain reservoirs for further irrigation usage while channel irrigation is to drain water directly from the river for irrigation without storage in a reservoir. There are two main reservoirs which were built for irrigation purpose. The Duolang Reservoir^45^ is located at 40 km, northwest to the Alar city and consists of an old reservoir built in 1965 and a new reservoir built in 1995. It is worth mentioning that the storage capacity turned to 43 million m^3^ after the expansion in 1971 and total reservoir capacity is approximately 120 million m^3^ since 1995. The Shengli Reservoir^46^, located about 30 km, west to the Alar city, was built in 1965 with a capacity of approximately 108 million m^3^, and it began to storage water by the end of 1970. In the upper reach of the Xinquman station, several water diversion irrigation projects were built during 1989–1994. Note that the water diversion projects were not operated in a well-controlled manner according to the data from the Tarim River Basin management Bureau^47^.

### Data

The ArcGIS 10.1 and downloaded DEM data (http://www.gscloud.cn/) of the Tarim Basin with a resolution of 90 m were used to extract drainage network (Fig. 8). Daily streamflow data collected from the two representative hydrological stations, the Alar station and the Xinquman station on the mainstream of the Tarim Basin during 1957–2014 were analyzed, in addition to the construction years of water diversion irrigation projects from 1990 through 1994. Based on operation time of the plain reservoirs and the water diversion irrigation projects, the streamflow data were divided into three periods: the pre-impact period (from 1957 through 1972), representing impact of natural flow regimes; the reservoir irrigation (RI) impact period (from 1973 through 1989) for water diversion from the mainstream of the Tarim River mainly transferred to plain reservoirs, and the channel irrigation (CI) period (from 1995 through 2014).

## Methods

The set of indicators of hydrological alteration, initially proposed by Richter *et al*.^16^ are used in this study to assess impacts of human activities (reservoir irrigation and channel irrigation) on flow regimes of the Tarim River. The IHAs are categorized into 5 groups for characterizing a flow regime in terms of magnitude of monthly flow, magnitude and duration of annual extreme flows and the base flow condition, timing of annual extreme flow conditions, frequency and duration of high and low pulses, and rate and frequency of flow changes (Table 5). Based on initial screening of daily streamflow data from the Alar station and the Xinquman station, the indicator of zero flow days is excluded and therefore only 32 indicators are assessed for characterization of the flow regime in the Tarim River.

**Table 5:** Indicators of hydrological alteration (32 IHAs).

Group	Group description	Hydrological indicators
Group 1 (12 IHAs)	Magnitude of monthly flow	Average flow of each calendar month
Group 2 (11 IHAs)	Magnitude and duration of annual extreme flows, and the base flow condition	Annual minimum 1-, 3-, 7-, 30-, 90-day means Annual maximum 1-, 3-, 7-, 30-, 90-day means Base flow index
Group 3 (2 IHAs)	Timing of annual extreme flow conditions	Julian date of annual 1-day minimum Julian date of annual 1-day maximum
Group 4 (4 IHAs)	Frequency and duration of high and low pulses	Number of low pulses each year. Mean duration of low pulse with each year. Number of high pulses each year. Mean duration of high pulse with each year
Group 5 (3 IHAs)	Rate and frequency of flow changes	Rise rate. Fall rate. Number of flow reversals

To quantitatively compare impacts of human activities on IHAs, each IHA is calculated in terms of median value, deviation degree, and degree of hydrological alteration at the three periods: the pre-impacted, the reservoir irrigation (RI) impacted and channel irrigation (CI) impacted. The deviation degree for an IHA is calculated with the following equation,

1 $${P}_{i}=\frac{{M}_{e}-{M}_{o}}{{M}_{o}}\times 100 \% $$

where *M*_*o*_ and *M*_*e*_ are the median value for the pre-impacted and the post-impacted period which refers to either RI impacted or CI impacted. A positive *P*_*i*_ indicates an increased median value in the post-impacted period compared to the pre-impacted period while a negative *P*_*i*_ suggests a decreased median value in the post-impacted period compared to the pre-impacted period. Degree of hydrological alteration of a flow regime can be further calculated for each indicator according to the following equation^15^,

2 $${D}_{i}=|\frac{{N}_{o}-{N}_{e}}{{N}_{e}}|\times 100 \% $$

where *N*_*o*_ is the observed number of post-impacted years for which the value of the indicator falls within the RVA target range, from 25^th^ percentile to 75^th^ percentile, as suggested by Richter *et al*.^15^. *N*_*e*_ is the expected number of post impacted years for which the value of indicator falls within the targeted range and can be estimated by *r × N*_*T*_ (r is percentage of pre-impacted years for which the value of an indicator falls within the RVA target range, and N_T_ is total number of post impacted years). Generally, RVA presumes natural flow series of a flow regime to be an ideal condition. If an environmental flow scheme attains a preset target range at a same frequency as that occurred naturally, then the flow regime is expected to be healthy. It is suggested that *D*_*i*_ < 33% for little or no alteration, 33% < *D*_*i*_ < 67% for moderate alteration, and *D*_*i*_ > 67% for high alteration^15^. However, Equation (2) is used to estimate *D*_*i*_ for each of 32 indicators. Some _*i*_ndicators may show low alteration in a flow regime while some other may have a highly degree of alteration. An overall degree of hydrological alteration for all 32 indicators can be calculated according to the following equation,

3 $$D=\sqrt{\frac{{\sum }_{i=1}^{32}{D}_{i}^{2}}{32}}$$

One limitation to use Equation (3) for calculating the overall degree of hydrological alteration is that the impact of an indicator having highly degree of alteration on the flow regime could be easy underestimated among most indicators having a moderate (or low) degree of alteration. In order to overcome the limitation in Equation (3), an improved overall degree of hydrologic alteration is calculated according to the following equation,

4 $$D=\sqrt{\frac{{D}_{jmax}^{2}+{D}_{w}^{2}}{2}}$$

where *D*_*jmax*_ and *D*_*w*_ are the maximum and average values of degree of alteration for the each group of indicators. In this study, the impacts of water diversion projects on the flow regime in the Tarim River is evaluated based on the improved overall degree of alteration, estimated using Equation (4) and classified into 5 groups: slightly alteration, low alteration, moderation alteration, highly alteration and severe alteration(Table 6).

**Table 6:** Classification of the improved overall degree of hydrological alteration.

Grade	slight alteration	low alteration	moderate alteration	highly alteration	severe alteration
*D*	< 20%	20–40%	40–60%	60–80%	> 80%
